# Male circumcision and *Neisseria gonorrhoeae*, *Chlamydia trachomatis* and *Trichomonas vaginalis*: observations after a randomised controlled trial for HIV prevention

**DOI:** 10.1136/sti.2008.032334

**Published:** 2008-12-15

**Authors:** J Sobngwi–Tambekou, D Taljaard, M Nieuwoudt, P Lissouba, A Puren, B Auvert

**Affiliations:** 1 INSERM U687, Hôpital Paul Brousse, Villejuif, France; 2 Progressus, Johannesburg, South Africa; 3 National Institute for Communicable Diseases, Johannesburg, South Africa; 4 INSERM U687, Assistance Publique-Hôpitaux de Paris, University of Versailles, France

## Abstract

**Objective:**

To assess the association between male circumcision and *Neisseria gonorrhoeae*, *Chlamydia trachomatis* and *Trichomonas vaginalis* using data from a male circumcision randomised controlled trial.

**Methods:**

We used data collected during the male circumcision trial conducted in Orange Farm (South Africa) among men aged 18–24 years. Altogether, 1767 urine samples collected during the final follow-up visit were analysed using PCR. Prevalence of *N gonorrhoeae*, *C trachomatis* and *T vaginalis* was assessed as a function of male circumcision using odds ratios (OR) given by univariate and multivariate logistic regression.

**Results:**

In an intention-to-treat analysis, prevalence of *N gonorrhoeae*, *C trachomatis* and *T vaginalis* among intervention and control groups were 10.0% versus 10.3% (OR 0.97; p = 0.84), 2.1% versus 3.6% (OR 0.58; p = 0.065) and 1.7% versus 3.1% (OR 0.54; p = 0.062), respectively. The association between *T vaginalis* and male circumcision remained borderline when controlling for age, ethnic group, number of lifetime partners, marital status, condom use and HIV status (AOR 0.48; p = 0.069). In the as-treated analysis, this association became significant (OR 0.49, p = 0.030; AOR 0.41, p = 0.030).

**Conclusions:**

This study demonstrates for the first time that male circumcision reduces *T vaginalis* infection among men. This finding explains why women with circumcised partners are less at risk for *T vaginalis* infection than other women. The protective effect on *T vaginalis* is an additional argument to recommend male circumcision in Africa where it is acceptable.

**Trial registration number:**

NCT00122525.

Recent evidence has shown that male circumcision is a promising prevention approach for sexually transmitted infections (STIs): three randomised controlled trials (RCTs)^1^^–^3 have shown that male circumcision reduces HIV infection among young men in Africa. According to a meta-analysis published in 2006, circumcised men may be at lower risk of herpes simplex virus 2 (HSV-2) infection, chancroid and syphilis.^4^ However, there are conflicting results about the association of male circumcision and non-ulcerative STIs such as *Neisseria gonorrhoeae*, *Chlamydia trachomatis*^5^^–^11 and *Trichomonas vaginalis* infections among men.^12^^–^14

Infection with non-ulcerative STIs is major public health issue. There are about 62 million new cases of *N gonorrhoeae* annually worldwide, with an estimated incidence of 17 million in sub-Saharan Africa.^15^ *N gonorrhoeae* is asymptomatic in only 10% of men but primarily asymptomatic in women and its complications can be lethal.^15^ *C trachomatis* worldwide incidence has been estimated at 92 million annually, with about 16 million occurring in sub-Saharan Africa.^15^ It is a significant public health concern because *C trachomatis* infection is asymptomatic in over 50% of cases among men and women^15^ and it can lead to serious health complications if untreated.^16^ Finally, *T vaginalis* is the most common non-viral STI in the world, with 174 million new cases estimated in 1999.^15^ In sub-Saharan Africa, the incidence is estimated at 32 million.^15^ The infection is asymptomatic in about 50% of infected women and in over 90% of men;^15^ thus, re-infection and re-exposure is problematic.^17^ Furthermore, co-infections among these three STIs are common.^18^ ^19^

The objective of this study was to analyse the effect of male circumcision on *N gonorrhoeae, C trachomatis* and *T vaginalis* prevalence using data collected during a male circumcision RCT conducted in Orange Farm, South Africa.^1^

## METHODS

### Collection of data

The technical details of the trial (ANRS-1265 study), including the description of the population, has been published elsewhere.^1^ Between February 2002 and July 2004, 3274 uncircumcised male volunteers, aged 18 to 24 years, signed a consent form and were recruited, randomised into two groups and followed-up. Male circumcision was offered immediately after randomisation to the intervention group and after the end of the follow-up period to control group participants. During each follow-up visit at 3, 12 and 21 months, circumcision status was assessed by a nurse through genital examination, a blood sample was taken and information about sexual behaviour was collected.

For 318 consecutive days, between 10 January 2005 and 24 November 2005, a 10 ml sample of first-voided urine was collected from all participants coming for the 21-month visit during this period. These samples were analysed to assess the association between male circumcision and *N gonorrhoeae, C trachomatis* and *T vaginalis* prevalence.

### Laboratory methods

Urine samples were frozen at −20°C immediately after collection and kept frozen until processing. *N gonorrhoeae* and *C trachomatis* testing was performed using the COBAS Amplicor detection kit (Roche Molecular Diagnostics, Pleasanton, California, USA). For the detection of *T vaginalis*, a qualitative FRET-based real-time PCR diagnostic test (Roche Molecular Diagnostics) was used based on previous published literature and validated using characterised specimens.^20^ ^21^ PCR has been shown to identify significantly more trichomoniasis cases than culture.^22^

### Data analysis

Categorical data of the control and intervention groups were compared using χ^2^ or Fisher exact test when appropriate and numerical data were compared using the Kruskal-Wallis test. *N gonorrhoeae, C trachomatis* and *T vaginalis* positive samples were analysed using intention-to-treat and as-treated analyses with univariate logistic regression. These analyses were repeated multivariately to control for ethnic group, education, age, number of lifetime partners, marital status, condom use and HIV status. To assess whether the effect of male circumcision on *T vaginalis* was independent of HIV infection, which is reduced by male circumcision^1^^–^3 and associated with *T vaginalis* infection,^23^ the analysis of the association between *T vaginalis* and male circumcision was repeated a) among those who remained HIV negative during follow-up and b) excluding those who were HIV positive at recruitment. To evaluate the effect of an imbalance between the groups, analyses of significant results were repeated when controlling for the propensity score coded in quintiles.^24^

## RESULTS

The baseline characteristics of the participants who were tested for *C trachomatis*, *N gonorrhoeae* and *T vaginalis* by randomisation group are reported in table 1. The characteristics of those who did not attend follow-up visits during which biological samples were collected but who came for the last follow-up visit are also reported in table 1. Randomisation groups differed according to their ethnic distribution, the number of sex acts and HIV status. When compared with study participants, those not tested for *C trachomatis*, *N gonorrhoeae* and *T vaginalis* had a higher number of lifetime partners and a higher HIV prevalence.

**Table 1:** Background characteristics, reported sexual behaviour and HIV prevalence at the 21-month visit

	Control	Intervention*	All participants tested (control + intervention)	Participants not tested for CT, NG and TV†
	n = 881	n = 886 (p value)	n = 1767*	n = 1188 (p value)
Background characteristics				
Ethnic group				
Sotho	53.0%	54.0% (0.012)	53.5%	40.6% (<0.001)
Zulu	33.52%	28.3%	30.9%	42.3%
Other	13.5%	17.7%	15.6%	17.1%
<21 years old	33.3%	29.1% (0.065)	31.2%	32.9% (0.33)
Primary level of education completed	98.9%	98.0% (0.18)	98.4%	98.1% (0.48)
Married or living as married§	4.7%	5.7% (0.45)	5.2%	7.1% (0.061)
Reported sexual behaviour				
Mean (median) number of lifetime sex partners	4.2 (4.0)	4.4 (4.0) (0.55)	4.3 (4.0)	4.8 (4.0) (<0.001)
Mean (median) number of non-spousal sex partners‡	0.88 (1.0)	0.94 (1.0) (0.48)	0.91 (1.0)	0.87 (1.0) (0.73)
Mean (median) number of sex acts‡	7.4 (2.0)	9.0 (3.0) (0.045)	8.2 (3.0)	7.0 (3.0) (0.65)
Consistent condom use with non-spousal sex partners‡¶	23.4%	24.6% (0.70)	24.1%	5.2% (<0.001)
HIV prevalence				
HIV positive	7.1%	4.5% (0.025)	5.8%	8.0% (0.02)

*The p value corresponds to the comparison of the control and intervention group; †the p value corresponds to the comparison of those tested for *Neisseria gonorrhoeae* (NG)*, Chlamydia trachomatis* (CT) and *Trichomonas vaginalis* (TV) with those not tested; ‡during the past 12 months; §at some time during the past 12 months; ¶among those having had sexual intercourse during the past 12 months.

Tables 2–4 present the univariate and multivariate association between male circumcision and *N gonorrhoeae, C trachomatis* and *T vaginalis*, respectively. It was found that there was no effect of male circumcision on *N gonorrhoeae,* as demonstrated by the odds ratio (OR) values close to 1 in table 2. The borderline association between male circumcision and *C trachomatis* in the intention-to-treat analysis disappeared in the as-treated analysis. The borderline association between male circumcision and *T vaginalis* in the intention-to-treat analysis became significant in the univariate and multivariate as-treated analysis. The adjusted ORs were slightly lower than the corresponding univariate ORs with values close to 0.5.

**Table 2:** Association between *Neisseria gonorrhoeae* (NG) prevalence and male circumcision

	NG prevalence % (positive/total)	OR (95% CI; p value)	AOR* (95% CI; p value)
Randomisation group			
Control	10.3% (91/881)	1	1
Intervention	10.0% (89/886)	0.97 (0.71 to 1.32; p = 0.84)	0.94 (0.69 to 1.29; p = 0.72)
Circumcision status			
Uncircumcised	10.0% (88/878)	1	1
Circumcised	10.4% (92/887)	1.04 (0.76 to 1.41; p = 0.81)	1.02 (0.74 to 1.40; p = 0.91)

*Adjusted odds ratio on ethnic group, age, education, number of lifetime partners, marital status, condom use and HIV status.

**Table 3:** Association between *Chlamydia trachomatis* (CT) prevalence and male circumcision

	CT prevalence % (positive/total)	OR (95% CI; p value)	AOR* (95% CI; p value)
Randomisation group			
Control	3.6% (32/881)	1	1
Intervention	2.1% (19/886)	0.58 (0.33 to 1.03; p = 0.065)	0.56 (0.32 to 1.00; p = 0.052)
Circumcision status			
Uncircumcised	3.3% (29/878)	1	1
Circumcised	2.5% (22/887)	0.74 (0.42 to 1.31; p = 0.30)	0.75 (0.42 to 1.32; p = 0.31)

*Adjusted odds ratio on ethnic group, age, education, number of lifetime partners, marital status, condom use and HIV status.

**Table 4:** Association between *Trichomonas vaginalis* (TV) prevalence and male circumcision

	TV prevalence % (positive/total)	OR (95% CI; p value)	AOR* (95% CI; p value)
Randomisation group			
Control	3.1% (27/881)	1	1
Intervention	1.7% (15/886)	0.54 (0.29 to 1.03; p = 0.062)	0.53 (0.28 to 1.02; p = 0.056)
Circumcision status			
Uncircumcised	3.2% (28/878)	1	1
Circumcised	1.6% (14/887)	0.49 (0.25 to 0.93; p = 0.030)	0.47 (0.25 to 0.92; p = 0.027)

*Adjusted odds ratio on ethnic group, age, number of lifetime partners, marital status, condom use and HIV status, excluding education because of the limited number of cases.

When excluding those who HIV seroconverted during follow-up, the OR values reported in table 4 remained almost unchanged with relative variation between −7.3% and +3.7% (results not shown). This indicates that the effect of male circumcision on *T vaginalis* is independent of the effect of male circumcision on HIV.

When excluding those who were HIV positive at recruitment, the OR values and p values reported in table 4 became slightly lower. The OR and adjusted OR (AOR) associated with randomisation groups were 0.45 (95% CI 0.22 to 0.89; p = 0.023) and 0.39 (95% CI 0.19 to 0.80; p = 0.0098), respectively. The OR and AOR associated with circumcision status were 0.34 (95% CI 0.14 to 0.82; p = 0.016) and 0.39 (95% CI 0.18 to 0.76; p = 0.065), respectively. The AORs of table 4 were almost identical when the analyses were adjusted for the propensity score in addition to the other covariates.

## DISCUSSION

This study demonstrates that male circumcision does not have a protective effect on *C trachomatis* acquisition in men, which concurs with the findings from most studies exploring this association whether assessed in cross-sectional studies^5^ ^8^ ^25^ ^26^ or in cohort studies.^8^^–^11 27 Only one multi-site study pooling cross-sectional data of 305 couples from Thailand, the Philippines, Brazil, Colombia and Spain found that when controlling for the number of sexual partners of the couple, male circumcision was associated with an 82% reduction in the risk of *C trachomatis* infection in female partners (OR 0.18, 95% CI 0.05 to 0.58). However, *C trachomatis* infection was not ascertained in the men themselves in that study and the authors admit that it is possible that male circumcision reduces the risk of transmission of the infection to the partner without reducing the risk of *C trachomatis* acquisition in the men themselves.^16^ In fact, two studies seem to suggest that *C trachomatis* prevalence is higher among circumcised men and their partners.^9^ ^27^

This study has some limitations. Biological samples were not collected throughout the follow-up period, so the *C trachomatis*, *N gonorrhoeae* and *T vaginalis* statuses at inclusion are unknown. As a result, some *T vaginalis* infections may have predated the intervention. Thus, we report the effect of male circumcision on *T vaginalis* prevalence and not *T vaginalis* incidence. Only participants coming for the last follow-up visit and during a specified time period were tested for *C trachomatis*, *N gonorrhoeae* and *T vaginalis*. This may have introduced some bias. Indeed, we found that those having undergone STIs testing were slightly different from those who had not. However, this difference was not expected to change the association between male circumcision and the *C trachomatis*, *N gonorrhoeae* and *T vaginalis* statuses. Lastly, the slight difference between circumcised and uncircumcised participants, which may be partly explained by a differential follow-up, may also have interfered with the result of this study. Hence, the fact that the results were not changed when adjusting on the propensity score is reassuring. Nevertheless, the results of this study have to be confirmed using the data of the two other male circumcision trials conducted in Kenya and Uganda.^2^ ^3^

No evidence of a protective effect of male circumcision on *N gonorrhoeae* infection was found. Previous studies have suggested that results will vary according to the population assessed: four studies among male attendees of STI clinics in developed countries found that uncircumcised men were up to twice as likely to develop *N gonorrhoeae* infection than circumcised men (OR of 1.6 to 2.0).^7^ ^8^ ^26^ ^28^ However, none of the studies conducted in developing countries found evidence of such an effect.^6^ ^9^ ^27^ ^29^

The study demonstrated a borderline protection effect of male circumcision on *T vaginalis* infection by young men in the intention-to-treat analysis and a significant effect in the as-treated analysis. The difference between the two analyses may have been caused by the high proportion of cross-over in this RCT,^1^ which diluted the effect observed in the intention-to-treat analysis. The fact that the protective effect became slightly stronger in the multivariate analysis, which includes HIV status, also suggests a protective independent effect of male circumcision on *T vaginalis* acquisition.

This finding is noteworthy because very few studies have investigated this association in men—probably due to diagnostic limitations.^14^ ^17^^–^19 Nevertheless, the size of the protective effect obtained in this study is consistent with what has been estimated by observational studies. A cross-sectional investigation conducted among men from the general population of Mwanza, Tanzania, found that male circumcision status was significantly associated with *T vaginalis* infection (OR 0.37, 95% CI 0.19 to 0.72) when adjusting for age.^12^ In their prospective study among US male partners of women infected with *T vaginalis*, Seña and colleagues found that uncircumcised men were almost twice as likely to be infected with *T vaginalis* (unadjusted OR 1.8, 95% CI 1.10 to 3.20).^14^

The fact that *N gonorrhoeae* and *C trachomatis* are almost exclusively urethral pathogens may explain why male circumcision has no protective effect against them. In contrast, the protective effect against *T vaginalis* may indicate that *T vaginalis* is both a sub-preputial and a urethral pathogen.

There is also evidence that male circumcision reduces *T vaginalis* acquisition by female partners. A recent randomised study conducted in Rakai, Uganda, among HIV discordant heterosexual couples indicated that the rate of *T vaginalis* infection among partners of participants from the intervention arms was reduced by almost half (adjusted risk ratio 0.52, 95% CI 0.05 to 0.98).^13^ Hence, our study illustrates why male circumcision is protective against *T vaginalis* infection among women having circumcised partners. Indeed, as shown in our study, male circumcision reduces the risk of *T vaginalis* infection among men and consequently reduces the exposure of women to *T vaginalis*. Thus, the risk of *T vaginalis* infection is lowered among women.

Some studies have suggested that *T vaginalis* facilitates the spread of HIV by up to twofold.^15^ ^23^ ^30^ ^31^ Thus, the effect of male circumcision on HIV acquisition in young men may partly be due to its effect on *T vaginalis*. If the results of this study are confirmed by those of the male circumcision trials conducted in Uganda and Kenya, the findings of this study will reinforce the WHO-UNAIDS statement recommending the implementation of male circumcision programmes in African countries with low male circumcision prevalence and a high male circumcision acceptability.^32^

**Key messages**

- Male circumcision protects men against *Trichomonas vaginalis* infection, which is the most common non-viral sexually transmitted infection in the world.
- *T vaginalis* infection causes severe morbidity among women. Male circumcision indirectly benefits women by reducing their exposure to *T vaginalis*.
- Male circumcision does not provide protection against *Neisseria gonorrhoeae* or *Chlamydia trachomatis*.
